# The application of health information technology for the elderly care in the emergency department: a conceptual model

**DOI:** 10.1186/s12877-024-05212-w

**Published:** 2024-07-19

**Authors:** Ghazal Shagerdi, Haleh Ayatollahi, Morteza Hemmat, Kimia Zeraatkar

**Affiliations:** 1 Department of Health Information Management, School of Health Management and Information Sciences, Iran University of Medical Sciences, Tehran, Iran; 2 Health Management and Economics Research Center, Health Management Research Institute, Iran University of Medical Sciences, Tehran, Iran; 3 Department of Health Information Technology, Saveh University of Medical Sciences, Saveh, Iran; 4 Education Development Center, Hormozgan University of Medical Sciences, Bandar Abbas, Iran

**Keywords:** Elderly, Elderly care, Emergency department, Emergency medical services, Health information technology

## Abstract

**Introduction:**

In the emergency departments (EDs), usually the longest waiting time for treatment and discharge belongs to the elderly patients. Moreover, the number of the ED admissions for the elderly increases every year. It seems that the use of health information technology in geriatric emergency departments can help to reduce the burden of the healthcare services for this group of patients. This research aimed to develop a conceptual model for using health information technology in the geriatric emergency department.

**Methods:**

This study was conducted in 2021. The initial conceptual model was designed based on the findings derived from the previous research phases (literature review and interview with the experts). Then, the model was examined by an expert panel (*n* = 7). Finally, using the Delphi technique (two rounds), the components of the conceptual model were reviewed and finalized. To collect data, a questionnaire was used, and data were analyzed using descriptive statistics.

**Results:**

The common information technologies appropriate for the elderly care in the emergency departments included emergency department information system, clinical decision support system, electronic health records, telemedicine, personal health records, electronic questionnaires for screening, and other technologies such as picture archiving and communication systems (PACS), electronic vital sign monitoring systems, etc. The participants approved all of the proposed systems and their applications in the geriatric emergency departments.

**Conclusion:**

The proposed model can help to design and implement the most useful information systems in the geriatric emergency departments. As the application of technology accelerates care processes, investing in this field would help to support the care plans for the elderly and improve quality of care services. Further research is recommended to investigate the efficiency and effectiveness of using these technologies in the EDs.

**Supplementary Information:**

The online version contains supplementary material available at 10.1186/s12877-024-05212-w.

## Introduction

Aging is an important part of the natural human life, which has recently caused new challenges for the health systems [1]. The elderly are the most recipients of healthcare services and contribute to increase emergency department (ED) visits [2]. It should be noted that the number of the elderly admitted to the EDs increases annually, and they spend more time in this department than other age groups [2–4]. However, the high workload and the lack of adequate staff in the EDs may cause challenges in the provision of high-quality care to the elderly [5]. In addition, overcrowding in hospitals may reduce the quality of care, increase medical errors, decrease patient satisfaction, and create a repetitive cycle of hospitalization [6]. Considering the complexity of the elderly care and their unique needs in the EDs, it seems that the use of various types of health information technologies in this department can improve data collection processes, quality of care, and access to the elderly health data [7].

Health information technologies have a number of potentials to improve quality, safety, and efficiency of patient care in different fields including geriatric emergency medicine [8–10]. These technologies help to improve treatment outcomes, reduce mortality, support emergency care services [11, 12], and improve clinical decision making processes [11, 13] through integrating various types of information systems used in pre-hospital and hospital emergency care services [11, 14–18]. Some of these technologies are telemedicine, electronic health records, clinical decision support systems, web-based packages for patients and their families, and assistive information technologies in the EDs, especially for the elderly [19, 20].

In Iran, population aging is rapidly increasing [21] and it is estimated that the ratio of the elderly to the population will be 14.5% within the next 15 years and 22% within the next 25 years. This will increase economic pressure on the active population of the country [22]. Moreover, providing emergency care services for such a large group of patients may not be very effective [23]. According to numerous studies, the inadequacy of documentation in the EDs [23–28], illegibility of emergency care records [29, 30], and the high percentage of medication errors in the EDs especially for patients aged from 50 to 75 years old, expose the elderly to the consequences of the prolonged stay in the EDs [25].

In addition, due to the limited financial and physical resources in the field of geriatric care [31, 32], it is necessary to use alternative solutions to improve the quality of care. The results of the previous studies on the use of various geriatric emergency care models indicated that it is necessary to formulate new practical models with interdisciplinary features to address the healthcare requirements of the elderly, save costs, and reduce patient re-admission rate [33, 34]. According to a literature review, although recent developed health information technologies, including health information systems, computerized provider order entries, and electronic health records have reduced challenges in providing elderly care services in the EDs, there are still many opportunities to help these patients [35]. The experts in the field of geriatric emergency medicine also believed that the use of health information technologies in the emergency departments can lead to a greater focus on the optimal use of the tresources for the elderly care, and improve emergency care services for them [36]. Therefore, this study aimed to develop a conceptual model for using health information technology in the geriatric emergency department.

## Methods

This was a mixed-methods study conducted in Iran in 2021. Before conducting the research, the ethics approval was obtained from the university ethics committee. Initially, a review study was conducted by the research team members (GS, HA, MH) to identify the application of various types of health information technologies for the elderly in emergency care services [35]. In the second phase of the research, opportunities for using various health information technologies for the elderly care in the emergency departments were investigated. In this phase, 33 experts in the fields of geriatric medicine, geriatric nursing, and emergency medicine were interviewed. These individuals lived in the capital of Iran (Tehran) and worked either in the emergency departments or in the medical universities [36]. Then, an initial conceptual model was designed based on the findings derived from the first and the second phases of the study, and was examined by an expert panel (*n* = 7). Finally, using the Delphi technique (two rounds), the components of the conceptual model were approved and finalized by more experts. In this paper, the results of the Delphi study are reported.

### Participants

The purposive sampling method was used to select the eligible participants. The potential participants included the faculty members of the geriatric medicine departments of two medical universities and the faculty members of the geriatric nursing departments of four medical universities. Other participants were the emergency medicine specialists and nurses who worked in three different university hospitals. In total, 223 individuals were found eligible to participate in the research. All of the participants had at least three years of work experience in the field of emergency or geriatric medicine/nursing. These people were invited to complete the research questionnaire either in a personal meeting with one of the researchers (GS) or after receiving the questionnaire via emails.

### Research instrument

A 90-item questionnaire was designed based on the literature review and findings derived from previous stages of the research [35, 36]. It was a five-point Likert scale questionnaire ranged from very important (5), important (4), moderately important (3), slightly important (2), and not important (1) (Appendix I). The face and content validity of the questionnaire were assessed by six faculty members of geriatric medicine, emergency medicine, and geriatric nursing. The questionnaire was piloted before being sent to the actual research participants. It was completed by 10 emergency medicine specialists and nurses, who were out of the research sample, to ensure that it is appropriate for collecting the main data. The participants’ feedback helped to improve the readability of the questionnaire. The questionnaire for the first round of the Delphi study consisted of eight parts, participants’ demographic data (7 questions), applications of the emergency department information system (14 questions), clinical decision support system (8 questions), electronic health records (20 questions), telemedicine (14 questions), personal health records (19 questions), electronic questionnaire (5 questions), and other technologies (e.g., picture archiving and communication system (PACS), electronic vital sign monitoring systems, etc.) (1 question). An open-ended question was also considered to ask the participants about other useful health information technologies in the field of geriatric emergency medicine.

### Data analysis

Initially, Shapiro-Wilk test was performed to determine the normality of data distribution (*P* > 0.05). Then, descriptive statistics were used to analyze the data. If 75% of the participants or more chose the first two response options for an item (i.e., very important and important), and the mean value was more than 3.75, the item was considered important and entered into the final model. Those items for which a total of 50 to 75% of the participants chose the first two response options and their mean values were between 2.5 and 3.75, were asked again in the second round of the Delphi study. The items that were chosen by less than 50% of the participants and their mean values were less than 2.5, were considered less important from the experts’ point of views and were removed from the final model. In both rounds of the Delphi study, the same procedures were undertaken to analyze the data.

## Results

As Table 1 shows, in total 39 participants took part in the 1st round of the Delphi study. In the 2nd round, the total number of the participants was 18. Most of the participants were female in the first (*n* = 26, 66.7%) and second (*n* = 12, 66.6%) rounds of the Delphi study, and the highest frequency belonged to the participants aged over 40 years old. Most of the participants were nurses who had a bachelor’s degree. Although in the first round of the Delphi study, the highest frequency of work experience (*n* = 16, 41.1%) was more than 15 years, in the second round of the study, the work experience of 6 to 10 years (*n* = 6, 33.4%) had the highest frequency. The participants’ characteristics in both rounds of the Delphi study are presented in Table 1.

**Table 1:** Participants’ characteristics in the first and second rounds of the Delphi study

Variable	Participants	First round		Second round	
		Frequency	Percent	Frequency	Percent
Sex	Female	26	66.7	12	66.6
	Male	13	33.3	6	33.4
Age	*≤* 30	3	7.7	2	11.1
	31–40 years	14	35.9	4	22.3
	> 40	22	56.4	12	66.6
Education	M.D.	10	25.6	5	27.8
	Ph.D.	8	20.5	3	16.7
	M.Sc.	7	17.9	1	5.5
	B.Sc.	14	36	9	50
Work experience	*≤* 5	5	12.8	2	11.1
	6–10	8	20.5	6	33.3
	11–15	10	25.6	3	16.7
	> 15	16	41.1	7	38.9
Job	Geriatric Medicine specialist	2	5.1	2	11.1
	Emergency Medicine specialist	8	20.5	3	16.7
	Faculty member (Nursing)	8	20.5	3	16.7
	Nurse (working in ED)	21	53.9	10	55.5

### 1st round of the Delphi study

Regarding the applications of the emergency department information system in the elderly care, the results indicated that most of the mentioned applications were approved by the experts (Table 2). Among them, the highest mean value was related to the clinical information documentation for the elderly patients (4.7 ± 0.4). The lowest mean value was related to the application of the system during ​​discharge to determine the length of stay in the emergency department (4.0 ± 0.8). This item did not reach a consensus and was asked again in the 2nd round of the Delphi study. In fact, out of 14 applications of the emergency department information system for the elderly care, 13 items were approved and one was left to the second round of the Delphi study.

**Table 2:** Applications of the emergency department information system for the elderly care in the emergency department

Number	Applications of emergency department information system		Very important Fr (%)	Important Fr (%)	Moderate importance Fr (%)	Less important Fr (%)	Unimportant Fr (%)	Mean ± SD	Median (1rd Q-3st Q)	Agreement
1	Data documentation	Personal information	26(66.6)	13(33.4)	0	0	0	4.6 ± 0.4	5(4–5)	✓
2		Clinical information	29(74.4)	10(25.6)	0	0	0	4.7 ± 0.4	5(4–5)	✓
3		Financial and insurance information	14(35.9)	18(46.2)	6(15.4)	1(2.5)	0	4.1 ± 0.7	4(4–5)	✓
4	Improving performance of the emergency department	Making clinical records available	23(59.0)	15(38.5)	1(2.5)	0	0	4.5 ± 0.5	5(4–5)	✓
5		Auditing emergency care	20(51.3)	17(43.6)	2(5.1)	0	0	4.4 ± 0.6	5(4–5)	✓
6		Extraction of evidence-based practice models	13(33.4)	19(48.7)	6(15.4)	1(2.5)	0	4.1 ± 0.7	4(4–5)	✓
7		Collaboration between the clinical staff in patient care	22(56.4)	16(41.1)	1(2.5)	0	0	4.5 ± 0.5	5(4–5)	✓
8		Providing quality and efficient care	24(61.6)	13(33.4)	1(2.5)	1(2.5)	0	4.5 ± 0.6	5(4–5)	✓
9		Identifying the challenges of caring for the elderly in the emergency department	19(48.7)	16(41.1)	4(10.2)	0	0	4.3 ± 0.6	4(4–5)	✓
10	Treatment	Documenting all types of procedures and interventions	27(69.3)	9(23.1)	3(7.6)	0	0	4.6 ± 0.6	5(4–5)	✓
11	Discharge	Documenting care plans after discharge	20(51.3)	14(35.9)	5(12.8)	0	0	4.3 ± 0.7	5(4–5)	✓
12		Documenting the date of the next visit	18(46.2)	15(38.5)	4(10.2)	2(5.1)	0	4.2 ± 0.8	4(4–5)	✓
13		Determining the length of stay in the emergency department	13(33.4)	14(35.9)	11(28.2)	1(2.5)	0	4.0 ± 0.8	4(3–5)	×
14		Follow-up after discharge to identify and meet the needs of the elderly	12(30.8)	21(53.9)	4(10.2)	2(5.1)	0	4.1 ± 0.7	4(4–5)	✓

✓ Agreement was reached × Agreement was not reached

Regarding the applications of the clinical decision support system in caring for the elderly in the emergency department, most of the applications were approved by the experts (Table 3). The highest mean value was related to the prevention of drug interactions (4.7 ± 0.4), and the lowest mean value was related to performing standard and necessary clinical screenings for the elderly (4.2 ± 0.8). The use of the clinical decision support systems to reduce elderly visits to the emergency department did not reach a consensus in the first round and was examined again in the second round of the Delphi study. In fact, out of eight applications of clinical decision support system in the elderly care, seven were approved and one entered to the second round of the Delphi study.

**Table 3:** Applications of clinical decision support systems for the elderly care in the emergency department

Number	Applications of clinical decision support systems		Very important Fr (%)	Important Fr (%)	Moderate importance Fr (%)	Less important Fr (%)	Unimportant Fr (%)	Mean ± SD	Median (1rdQ-3stQ)	Agreement
1	Comprehensive assessment of the elderly	Standard and necessary clinical screenings for the elderly	17(43.6)	15(38.5)	6(15.4)	0	1(2.5)	4.2 ± 0.8	4(4–5)	✓
2		Prevention of drug interactions	30(76.9)	6(15.4)	3(7.7)	0	0	4.6 ± 0.6	5(5–5)	✓
3		Clinical assessment	24(61.6)	11(28.2)	4(10.2)	0	0	4.5 ± 0.6	5(4–5)	✓
4		Risk assessment of high risk patients	23(58.9)	13(33.4)	3(7.7)	0	0	4.5 ± 0.6	5(4–5)	✓
5	Improving performance of the emergency department	Focus on patient-centered care	21(53.8)	15(38.5)	3(7.7)	0	0	4.4 ± 0.6	5(4–5)	✓
6		Auditing emergency care	29(74.4)	7(18.0)	2(5.1)	1(2.5)	0	4.3 ± 0.8	4(4–5)	✓
7		Provision of quality and efficient care	23(59.0)	13(33.4)	2(5.1)	0	1(2.5)	4.4 ± 0.8	5(4–5)	✓
8	Treatment	Help to reduce elderly visits to the emergency department	22(56.5)	6(15.4)	9(23.1)	1(2.5)	1(2.5)	4.2 ± 1.0	5(3–5)	×

✓ Agreement was reached × Agreement was not reached

Regarding the applications of electronic health records, all proposed applications were approved in the first round of the Delphi study. Among the approved applications, “documenting all types of procedures and interventions” had the highest mean value (4.6 ± 0.58) and “follow-up after discharge to identify and meet the needs of the elderly”, “determining the length of stay in the emergency department”, and “extracting evidence-based practice models” had the lowest mean values (4.1 ± 0.7).

In terms of the applications of telemedicine in the elderly care, the results indicated that more than half of the proposed applications were approved by the experts (Table 4). The highest mean value was related to the standard and necessary clinical screenings for the elderly (4.2 ± 0.8) and the lowest mean value was related to the social assessment (4.0 ± 0.8). However, five items including clinical assessment, the elderly performance assessment, social assessment, continuity of care during the treatment period, focusing on the patient-centered care, and telecare and telemonitoring did not reach a consensus in the first round of the Delphi study and entered into the 2nd round. In fact, out of 14 telemedicine applications, eight were approved and six were examined again in the 2nd round of the Delphi study.

**Table 4:** Applications of telemedicine for the elderly care in the emergency department

Number	Applications of telemedicine		Very important Fr (%)	Important Fr (%)	Moderate importance Fr (%)	Less important Fr (%)	Unimportant Fr (%)	Mean ± SD	Median (1rdQ-3stQ)	Agreement
1	Comprehensive assessment of the elderly	Standard and necessary clinical screenings for the elderly	17(43.6)	14(36.0)	7(17.9)	1(2.5)	0	4.2 ± 0.8	4(4–5)	✓
2		Prevention of drug interactions	13(33.4)	18(46.1)	8(20.5)	0	0	4.1 ± 0.7	4(4–5)	✓
3		Clinical assessment	20(51.3)	9(23.1)	10(25.6)	0	0	4.2 ± 0.85	5(3–5)	×
4		Performance assessment	18(46.2)	11(28.2)	10(25.6)	0	0	4.2 ± 0.8	4(3–5)	×
5		Social assessment	14(35.9)	13(33.4)	11(28.2)	1(2.5)	0	4.0 ± 0.8	4(3–5)	×
6		Environmental assessment	15(38.5)	15(38.5)	8(20.5)	1(2.5)	0	4.1 ± 0.8	4(4–5)	✓
7	Improving performance of the emergency department	Continuity of receiving care services from a single doctor during the treatment period	20(51.3)	9(23.1)	9(23.1)	1(2.5)	0	4.2 ± 0.9	5(3–5)	×
8		Focus on the patient-centered care	11(28.2)	18(46.2)	10(25.6)	0	0	4.2 ± 0.8	5(3–5)	×
9		Elderly health foresight and support	18(46.2)	14(35.9)	7(17.9)	0	0	4.2 ± 0.7	4(4–5)	✓
10		Collaboration between the clinical staff in patient care	17(43.6)	17(43.6)	5(12.8)	0	0	4.3 ± 0.6	4(4–5)	✓
11		Provision of quality and efficient care	16(41.1)	18(46.1)	5(12.8)	0	0	4.2 ± 0.6	4(4–5)	✓
12	Treatment	Telecare and telemonitoring	14(35.9)	15(38.5)	8(20.5)	2(5.1)	0	4.0 ± 0.8	4(3–5)	×
13	Discharge	Transitional care (taking care of the patient during the transfer from the emergency department to the inpatient department or home)	19(48.8)	12(30.8)	6(15.3)	0	0	4.2 ± 0.9	4(4–5)	✓
14		In-home care	15(38.5)	17(43.6)	4(10.3)	5(1.2)	1(2.5)	4.1 ± 0.9	4(4–5)	✓
15		Follow-up after discharge to identify and meet the needs of the elderly	11(28.2)	19(48.8)	7(17.9)	5(1.2)	0	4.0 ± 0.8	4(4–5)	✓

✓ Agreement was reached × Agreement was not reached

Regarding the applications of personal health records for the elderly care in the emergency department, the findings indicated that most of these applications were approved by the experts. The highest mean value was related to the clinical assessment (4.6 ± 0.54) and the lowest mean value was related to the post-discharge follow-up to identify and meet the needs of the elderly (3.8 ± 1.0). As three items including “financial and insurance information”, “extraction of evidence-based practice models”, and “follow-up after discharge to identify and meet the needs of the elderly” did not reach an agreement in the first round of the study, they were asked again in the second round. In fact, out of 18 items related to the applications of personal health records for the elderly in the emergency department, 15 were approved and 3 were entered into the 2nd round of the Delphi study. The last section was related to the application of the electronic questionnaires for the elderly care in the emergency department, and clinical assessment (4.5 ± 0.5) had the highest mean value. The lowest mean value belonged to the environmental assessment (4.0 ± 0.7). All items of this section were approved by the experts in the first round of the Delphi study (Table 5). In addition, most of the participants agreed with the use of other health information technologies for the elderly care in the emergency department, especially for improving performance of this department (4.6 ± 0.6).

**Table 5:** Applications of electronic questionnaire for the elderly care in the emergency department

Number	Applications of electronic questionnaires		Very important Fr (%)	Important Fr (%)	Moderate importance Fr (%)	Less important Fr (%)	Unimportant Fr (%)	Mean ± SD	Median (1rdQ-3stQ)	Agreement
1	Comprehensive assessment of the elderly	Standard and necessary clinical screenings for the elderly	20(51.3)	15(38.5)	3(7.7)	1(2.5)	0	4.3 ± 0.7	5(4–5)	✓
2		Clinical assessment	23(59.0)	15(38.5)	1(2.5)	0	0	4.5 ± 0.5	5(4–5)	✓
3		Performance assessment	20(51.3)	18(46.2)	1(2.5)	0	0	4.4 ± 0.5	5(4–5)	✓
4		Social assessment	13(33.4)	20(51.3)	5(12.8)	1(2.5)	0	4.1 ± 0.7	4(4–5)	✓
5		Environmental assessment	11(28.3)	21(53.9)	6(15.3)	1(2.5)	0	4.0 ± 0.7	4(4–5)	✓

✓ Agreement was reached ×Agreement was not reached

### 2nd round of the Delphi study

The questionnaire of the 2nd round of the Delphi study included 11 items that did not reach an agreement by the experts in the first round. The questionnaire consisted of 18 items in two parts. The first part included the participants’ demographic data (7 questions) and the second part included, the applications of the emergency department information system (1 question), clinical decision support system (1 question), telemedicine (6 questions), and personal health records (3 questions) in caring for the elderly in the ED. In this round, all items were approved by the participants. Among these items, clinical assessment of the elderly using telemedicine had the highest mean value (4.6 ± 0.5), and documenting financial and insurance information of the elderly patients using personal health records had the lowest mean value (4.1 ± 0.7) (Table 6).

**Table 6:** The participants’ responses in the 2nd round of the Delphi study

Number	Health information technology	The application of health information technology for the elderly care in the emergency department		Very important Fr (%)	Important Fr (%)	Moderate importance Fr (%)	Less important Fr (%)	Unimportant Fr (%)	Mean ± SD	Median (1rdQ-3stQ)	Agreement
1	Emergency Department Information system	Discharge	Determining the length of stay in the emergency department	6 (33.4)	10 (55.5)	2 (11.1)	0	0	4.2 *±* 0.6	(5 − 4)4	✓
2	Clinical decision support system	Treatment	Help to reduce elderly visits to the emergency department	(50)9	7 (38.8)	2 (11.2)	0	0	4.3 *±* 0.7	4.5 (4–5)	✓
3	Telemedicine	Comprehensive assessment of the elderly	Clinical assessment	12 (66.7)	5 (27.8)	1 (5.5)	0	0	4.6 *±* 0.6	(5 − 4)5	✓
4			Performance assessment	12 (66.7)	3 (16.7)	3 (16.7)	0	0	4.5 *±* 0.8	(5 − 4)5	✓
5			Social assessment	7 (38.8)	8 (44.5)	3 (16.7)	0	0	4.2 *±* 0.7	(5 − 4)4	✓
6		Improving performance of the emergency department	Continuity of receiving care services from a single doctor during the treatment period	10 (55.7)	7 (38.8)	1 (5.5)	0	0	4.5 *±* 0.6	(5 − 4)5	✓
7			Focus on the patient-centered care	11 (61.2)	7 (38.8)	0	0	0	4.6 *±* 0.5	(5 − 4)5	✓
8		Treatment	Telecare and telemonitoring	11 (61.2)	(3/33)6	1 (5.5)	0	0	4.5 *±* 0.6	(5 − 4)5	✓
9	Personal health record	Documenting patients’ data	Financial and insurance information	6 (33.3)	8 (44.4)	4 (22.2)	0	0	4.1 *±* 0.7	4 (3.75-5)	✓
10		Improving performance of the emergency department	Extraction of the evidence-based practice models	7 (38.8)	10 (55.7)	1 (5.5)	0	0	4.3 *±* 0.6	(5 − 4)4	✓
11		Discharge	Follow-up after discharge to identify and meet the needs of the elderly	7 (38.8)	10 (55.7)	1 (5.5)	0	0	4.3 *±* 0.6	(5 − 4)4	✓

✓ Agreement was reached ×Agreement was not reached

## Discussion

In this study, the most common health information technologies used for the elderly care in the emergency department were identified and divided into seven general categories. These technologies included the emergency department information system, clinical decision support system, electronic health records, telemedicine, personal health records, electronic questionnaire, and other technologies such as the picture archiving and communication system, vital sign monitoring systems, etc.

In the previous studies, health information technologies used in the field of geriatric medicine were divided based on their applications [8–10]. For example, Vedel et al. [10] divided information technologies in the field of geriatric medicine into five main groups in terms of their applications: telecare technologies, electronic health records, clinical decision support systems, web-based systems for patients and their families, and assistive information technologies. In this study, all types of health information technologies for the elderly people were discussed, but their applications in the emergency department were not highlighted.

In another study, Schulz et al. [37] categorized the technologies used in the field of geriatric medicine into three groups, monitoring, diagnosis, and treatment, and into five dimensions of life, including physical and mental health, mobility, social relationships, safety, and daily activities and leisure time. The authors noted that information technology can be used in informing the elderly, providing necessary warnings, and increasing the speed of addressing their needs. Taheri et al. [38], divided the applications of information technology for the elderly health programs into 24 groups. However, the use of the technology was not investigated in the field of geriatric emergency medicine. Similary, Rosen et al. [39] identified four major categories for the application of mobile health technology in the field of geriatric medicine which included self-care, health care assistance, supervised health care, and continuous monitoring.

According to the results of the current research, emergency departement information system was one of the important systems that should be used for the elderly and other patients in the emergency departments, and most of the participants agreed upon its several applications. This system can help to collect patients’ clinical and non-clinical data that are important for developing care plans [40].

Another technology used for the elderly in the emergency department was clinical decision support system. These systems can help with increasing quality of care and safety, and using evidence-based medicine in practice [10]. Similarly, Vicente et al. [18] showed that the use of a pre-hospital clinical decision support system that allowed nurses to transfer the elderly directly to the emergency department was feasible and effective.

In terms of the electronic health records (EHR), a majority of the participants agreed with different applications of this system for the elderly in the emergency department. In fact, the electronic health records of the elderly provide a rich source of patient data that can be used by the clinical staff for providing heathcare services or can be used in patient self-management [10]. Bowles et al. [17] explained that geriatric nurse experts can efficiently influence patient outcomes and standardize older adults’ assessment and treatment by providing input into the decision support systems and EHRs. Similarly, Dowding et al. [41] found that EHR implementation was significantly associated with an increase in documentation rates for hospital acquired pressure ulcers (HAPU) risk, a 13% decrease in HAPU rates, but no decrease in fall rates.

Regarding telemedicine technology, the results suggested that more than half of the participants agreed with the applications of this technology for the elderly care in the emergency department. Telemedicine technology reduces the need for travel and facilitates access to the healthcare professionals. In addition, it can also help with saving time, and nurses can manage their time more efficiently [8–10]. Similarly, in Lim’s study [42], the results showed that the development of health information technologies, especially web-based technologies and wireless networks has led to significant improvements in telemonitoring of the elderly with chronic diseases. In another study, Morse et al. [43] conducted remote follow-up of the elderly patients through innovations in the geriatric emergency department.

The results of the current study also indicated that there might be other systems which can be used for the elderly care in the emergency department. Brunetti et al. [15] evaluated the benefits of remote medical diagnosis using a mobile-based electrocardiogram (ECG) for the elderly who requested emergency medical services. In another study, Brahmandam et al. [44] investigated the readiness and ability of the elderly to provide clinical information using a tablet in the emergency department. The results of this study revealed that the tablet might be a suitable tool for collecting clinical information of some elderly people in the emergency department; however, it will not be effective for a significant part of this population.

Overall, the results of the Delphi study in two rounds showed that all of the proposed systems and their applications were found important by the participants. This indicates that the findings of the previous studies worked well as building blocks of this study, and the results helped to design a conceptual model based on the overall consensus (Table 7).

**Table 7:** The model of using health information technology for the elderly care in the emergency department

Applications of health information technology for the elderly care in the emergency department		Health information technology						
		EHR	PHR	EDIS	E-Questionnaire	CDSS	Telemedicine	Other types of technology (PACS, etc.)
Documenting patient data	• Personal data	✓	✓	✓	×	×	×	×
	• Clinical data	✓	✓	✓	×	×	×	×
	• Financial data	✓	✓	✓	×	×	×	×
	• Insurance data	✓	✓	✓	×	×	×	×
Improving performance of the emergency department	• Availability of medical records	✓	×	✓	×	✓	×	×
	• Auditing emergency care	✓	✓	✓	×	✓	×	×
	• Extraction of evidence-based practice models	✓	✓	✓	×	×	×	×
	• Collaboration between clinical staff in patient care	✓	✓	✓	×	×	✓	×
	• Provision of quality and efficient care	✓	✓	✓	×	✓	✓	×
	• Identification of the elderly care challenges in the emergency department	✓	×	✓	×	×	×	×
	• Elderly health foresight and support	✓	✓	×	×	×	✓	×
	• Continuity of receiving care services from a single doctor during the treatment period	✓	✓	×	×	×	✓	×
	• Focus on the patient-centered care	✓	✓	×	×	✓	✓	×
Comprehensive assessment of the elderly	• Standard and necessary screening for the elderly	✓	✓	×	✓	✓	✓	×
	• Prevention of drug interactions	✓	✓	×	×	✓	✓	×
	• Clinical assessment	✓	✓	×	✓	✓	✓	×
	• Fall risk assessment	✓	×	×	×	×	×	×
	• Risk assessment of high risk patients	✓	✓	×	×	✓	×	×
	• Performance assessment	×	×	×	✓	×	×	×
	• Social assessment	×	×	×	✓	×	✓	×
	• Environmental assessment	×	×	×	✓	×	✓	×
Treatment	• Documenting all types of procedures and interventions)	✓	✓	✓	×	×	×	×
	• Help to reduce elderly visits to the emergency room	×	×	×	×	✓	×	×
	• Telecare and telemonitoring	×	×	×	×	×	✓	×
Discharge	• Documenting care plans after discharge	✓	✓	✓	×	×	×	×
	• Determining the date of the next visit	✓	✓	✓	×	×	×	×
	• Determining the length of stay in the emergency department	✓	✓	✓	×	×	×	×
	• Follow-up after discharge to identify and meet the needs of the elderly	✓	✓	✓	×	×	✓	×
	• Transitional care	×	×	×	×	×	✓	×
	• Home care	×	×	×	×	×	✓	×

According to this model, different clinical information systems such as electronic health records, telemedicine, and personal health records can be developed and used to optimize the elderly care in the emergency department [45]. This model helps healthcare practitioner, managers and other stakeholders to choose the most fit technology, and invest in implementing systems that can meet their requirements, particularly in the resource limited countries. A combination of different systems may also work well to support various aspects of emergency care services for the elderly. However, apart from the theoretical aspects, more studies are required to provide evidence on the efficiency and effectiveness of these systems in the emergency care services, especially for the elderly.

### Research limitations

One of the limitations of the current study was related to the limited number of the experts who completed the questionnaires. This might be due to the spread of the Covid-19 disease, the high workload of doctors and nurses who worked in the emergency departments, and the reluctance of some clinicians to take part in the research. Moreover, since most of the staff in the emergency departments were nurses, their participation in the first and second rounds of the Delphi study was more than other eligible individuals. Furthermore, the opinions of the elderly were not collected in this study. Future research can focus on investigating the opinions of the clinicians, elderly patients and their caregivers about using different types of information technologies for geriatric care in the emergency departments.

## Conclusion

This study was conducted to develop a conceptual model for using health information technology in the geriatric emergency department. The results of this study indicated that various types of health information technologies can be used in the processes of caring for the elderly in the ED. However, careful identification of opportunities and areas of application can help to better use of resources; improve staff performance, and quality of care services, particularly for this group of patients. In addition, complete, accurate, and integrated data collection regarding the elderly health status can be considered as a valuable resource for the training of other clinical staff. It is expected that the model presented in this study facilitates the process of choosing, developing and applying various types of health information technologies in the EDs and helps with conducting further research to determine how these systems can improve efficiency, effectiveness and quality of care for the elderly in the emergency departments.

### Electronic supplementary material

Below is the link to the electronic supplementary material.

Supplementary Material 1

## Data Availability

The datasets used and/or analyzed during the current study are available from the corresponding author on reasonable request.

## Abbreviations

ED: Emergency Department
PACS: Picture Archiving and Communication System
ECG: Electrocardiogram
HER: Electronic Heaath Records
HAPU: Hospital Acquired Pressure Ulcers
