# Supplementary material for: The application of health information technology for the elderly care in the emergency department: a conceptual model
Source: BMC Geriatr. 2024 Jul 19;24:618. doi: 10.1186/s12877-024-05212-w (PMC11264990; doi:10.1186/s12877-024-05212-w)
Supplement: Supplementary file 1 — Supplementary Material 1 [file 12877_2024_5212_MOESM1_ESM.docx]

Appendix I-Questionnaire

1. Participant’s characteristics
2. Age:
3. Sex: Male 🞎 Female 🞎
4. Field of study:
5. Name of the organization:
6. Level of Education: B.Sc. 🞎 M.Sc 🞎 Ph.D 🞎 M.D 🞎
7. Job position:
8. Work experience
9. Applications of health information technologies for the elderly care in the emergency department

| Number | Applications of emergency department information system | | Very important | Important | Moderate importance | Less important | Unimportant |
| --- | --- | --- | --- | --- | --- | --- | --- |
| 1 | Data documentation | Personal information |  |  |  |  |  |
| 2 |  | Clinical information |  |  |  |  |  |
| 3 |  | Financial and insurance information |  |  |  |  |  |
| 4 | Improving performance of the emergency department | Making clinical records available |  |  |  |  |  |
| 5 |  | Auditing emergency care |  |  |  |  |  |
| 6 |  | Extraction of evidence-based practice models |  |  |  |  |  |
| 7 |  | Collaboration between the clinical staff in patient care |  |  |  |  |  |
| 8 |  | Providing quality and efficient care |  |  |  |  |  |
| 9 |  | Identifying the challenges of caring for the elderly in the emergency department |  |  |  |  |  |
| 10 | Treatment | Documenting all types of procedures and interventions |  |  |  |  |  |
| 11 | Discharge | Documenting care plans after discharge |  |  |  |  |  |
| 12 |  | Documenting the date of the next visit |  |  |  |  |  |
| 13 |  | Determining the length of stay in the emergency department |  |  |  |  |  |
| 14 |  | Follow-up after discharge to identify and meet the needs of the elderly |  |  |  |  |  |

| Number | Applications of clinical decision support systems | | | | | | Very important | Important | | Moderate importance | | Less important | | | Unimportant | |
| --- | --- | --- | --- | --- | --- | --- | --- | --- | --- | --- | --- | --- | --- | --- | --- | --- |
| 15 | Comprehensive assessment of the elderly | | Standard and necessary clinical screenings for the elderly | | | |  |  | |  | |  | | |  | |
| 16 |  |  | Prevention of drug interactions | | | |  |  | |  | |  | | |  | |
| 17 |  |  | Clinical assessment | | | |  |  | |  | |  | | |  | |
| 18 |  |  | Risk assessment of high risk patients | | | |  |  | |  | |  | | |  | |
| 19 | Improving performance  of the emergency  department | | Focus on patient-centered care | | | |  |  | |  | |  | | |  | |
| 20 |  |  | Auditing emergency care | | | |  |  | |  | |  | | |  | |
| 21 |  |  | Provision of quality and efficient care | | | |  |  | |  | |  | | |  | |
| 22 | Treatment | | Help to reduce elderly visits to the emergency department | | | |  |  | |  | |  | | |  | |
| Applications of Electronic Health Records | | | | | | | | | | | | | | | | |
| 23 | Data documentation | | | | Personal information | |  |  | |  | |  | | |  | |
| 24 |  |  |  |  | Clinical information | |  |  | |  | |  | | |  | |
| 25 |  |  |  |  | Financial and insurance information | |  |  | |  | |  | | |  | |
| 26 | Comprehensive assessment of the elderly | | | | Standard and necessary clinical screenings for the elderly | |  |  | |  | |  | | |  | |
| 27 |  |  |  |  | Prevention of drug interactions | |  |  | |  | |  | | |  | |
| 28 |  |  |  |  | Clinical assessment | |  |  | |  | |  | | |  | |
| 29 |  |  |  |  | Risk assessment of high risk patients | |  |  | |  | |  | | |  | |
| 30 | Improving performance of the emergency department | | | | Making clinical records available | |  |  | |  | |  | | |  | |
| 31 |  |  |  |  | Continuity of receiving care services from a single doctor during the treatment period | |  |  | |  | |  | | |  | |
| 32 |  |  |  |  | Focus on the patient-centered care | |  |  | |  | |  | | |  | |
| 33 |  |  |  |  | Auditing emergency care | |  |  | |  | |  | | |  | |
| 34 |  |  |  |  | Elderly health foresight and support | |  |  | |  | |  | | |  | |
| 35 |  |  |  |  | Extraction of evidence-based practice models | |  |  | |  | |  | | |  | |
| 36 |  |  |  |  | Collaboration between the clinical staff in patient care | |  |  | |  | |  | | |  | |
| 37 |  |  |  |  | Provision of quality and efficient care | |  |  | |  | |  | | |  | |
| Number | | Applications of Electronic Health Records | | | | Very important | | | Important | | Moderate importance | | Less important | Unimportant | |  |
| 38 | | Treatment | | Documenting all types of procedures and interventions | |  | | |  | |  | |  |  | |  |
| 39 | | Discharge | | Documenting care plans after discharge | |  | | |  | |  | |  |  | |  |
| 40 | |  |  | Documenting the date of the next visit | |  | | |  | |  | |  |  | |  |
| 41 | |  |  | Determining the length of stay in the emergency department | |  | | |  | |  | |  |  | |  |
| 42 | |  |  | Follow-up after discharge to identify and meet the needs of the elderly | |  | | |  | |  | |  |  | |  |
| Applications of Telemedicine | | | | | | | | | | | | | | | |  |
| 43 | | Comprehensive assessment of the elderly | | Standard and necessary clinical screenings for the elderly | |  | | |  | |  | |  |  | |  |
| 44 | |  |  | Prevention of drug interactions | |  | | |  | |  | |  |  | |  |
| 45 | |  |  | Clinical assessment | |  | | |  | |  | |  |  | |  |
| 46 | |  |  | Performance assessment | |  | | |  | |  | |  |  | |  |
| 47 | |  |  | Social assessment | |  | | |  | |  | |  |  | |  |
| 48 | |  |  | Environmental assessment | |  | | |  | |  | |  |  | |  |
| 49 | | Improving performance of the emergency department | | Continuity of receiving care services from a single doctor during the treatment period | |  | | |  | |  | |  |  | |  |
| 50 | |  |  | Focus on the patient-centered care | |  | | |  | |  | |  |  | |  |
| 51 | |  |  | Elderly health foresight and support | |  | | |  | |  | |  |  | |  |
| 52 | |  |  | Collaboration between the clinical staff in patient care | |  | | |  | |  | |  |  | |  |
| 53 | |  |  | Provision of quality and efficient care | |  | | |  | |  | |  |  | |  |
| 54 | | Treatment | | Telecare and telemonitoring | |  | | |  | |  | |  |  | |  |
| 55 | | Discharge | | Transitional care (taking care of the patient during the transfer from the emergency department to the inpatient department or home) | |  | | |  | |  | |  |  | |  |
| 56 | |  |  | In-home care | |  | | |  | |  | |  |  | |  |
| 57 | |  |  | Follow-up after discharge to identify and meet the needs of the elderly | |  | | |  | |  | |  |  | |  |

| Number | Applications of Personal Health Records | | Very important | Important | Moderate importance | Less important | Unimportant |
| --- | --- | --- | --- | --- | --- | --- | --- |
| 58 | Data documentation | Personal information |  |  |  |  |  |
| 59 |  | Clinical information |  |  |  |  |  |
| 60 |  | Financial and insurance information |  |  |  |  |  |
| 61 | Comprehensive assessment of the elderly | Standard and necessary clinical screenings for the elderly |  |  |  |  |  |
| 62 |  | Prevention of drug interactions |  |  |  |  |  |
| 63 |  | Clinical assessment |  |  |  |  |  |
| 64 |  | Risk assessment of high risk patients |  |  |  |  |  |
| 65 | Improving performance of the emergency department | Continuity of receiving care services from a single doctor during the treatment period |  |  |  |  |  |
| 66 |  | Focus on the patient-centered care |  |  |  |  |  |
| 67 |  | Auditing emergency care |  |  |  |  |  |
| 68 |  | Elderly health foresight and support |  |  |  |  |  |
| 69 |  | Extraction of evidence-based practice models |  |  |  |  |  |
| 70 |  | Collaboration between the clinical staff in patient care |  |  |  |  |  |
| 71 |  | Provision of quality and efficient care |  |  |  |  |  |
| 72 | Treatment | Documenting all types of procedures and interventions |  |  |  |  |  |
| 73 | Discharge | Documenting care plans after discharge |  |  |  |  |  |
| 74 |  | Documenting the date of the next visit |  |  |  |  |  |
| 75 |  | Determining the length of stay in the emergency department |  |  |  |  |  |
| 76 |  | Follow-up after discharge to identify and meet the needs of the elderly |  |  |  |  |  |

| Number | Applications of electronic questionnaire | | Very important | Important | Moderate importance | Less important | Number |
| --- | --- | --- | --- | --- | --- | --- | --- |
| 77 | Comprehensive assessment of the elderly | Standard and necessary clinical screenings for the elderly |  |  |  |  |  |
| 78 |  | Clinical assessment |  |  |  |  |  |
| 79 |  | Performance assessment |  |  |  |  |  |
| 80 |  | Social assessment |  |  |  |  |  |
| 81 |  | Environmental assessment |  |  |  |  |  |
| Applications of other types of health information technologies (e.g., PACS, vital sign monitoring systems, etc.) | | | | | | | |
| 82 | Improving performance of the emergency department | Provision of quality and efficient care |  |  |  |  |  |
| 83 | If there are any other health information technologies used for the elderly care in the ED, please mention them and their applications. | | | | | | |
